# Extension of the Aggregation-Volume-Bias Monte Carlo Method to the Calculation of Phase Properties of Solid Systems: A Lattice-Based Cluster Approach

**DOI:** 10.1021/acs.jpca.2c04333

**Published:** 2022-08-08

**Authors:** Bin Chen

**Affiliations:** Department of Chemistry, Louisiana State University, Baton Rouge, Louisiana 70803-1804, United States

## Abstract

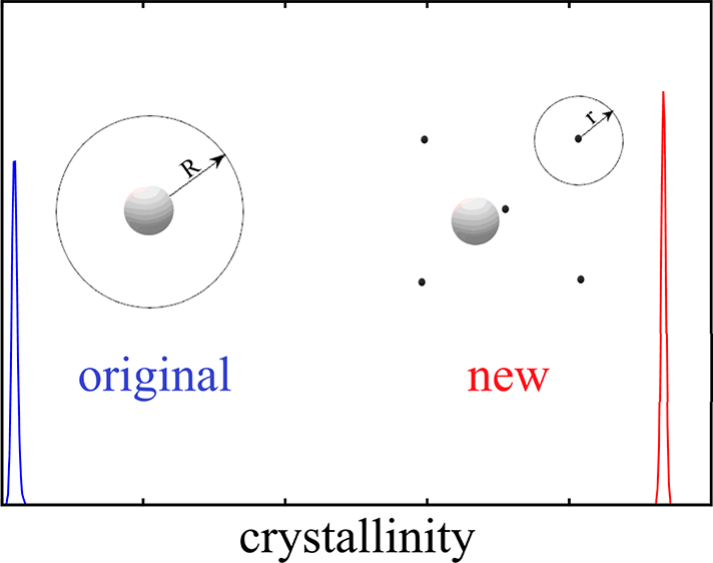

The aggregation-volume-bias Monte Carlo method, which has been successful in the calculation of the formation free energies of liquid clusters, is extended to solid systems. This extension is motivated by early studies where disordered clusters are observed when the original method is applied at a temperature even far below the triple point. In order to avoid the formation of disordered aggregates, the insertion of particles is targeted directly toward those crystal lattice sites. Specifically, the insertion volume used to be defined as a spherical volume centered around a given target molecule is now restricted to be around each of the crystal lattice sites near a given target molecule. The free energies obtained for both liquid and solid clusters are then used to extrapolate bulk-phase information such as the chemical potential of the liquid and solid phases at coexistence. Using the temperature and pressure dependencies of the chemical potential information obtained for both liquid and solid phases, the location of the triple point can be determined. For Lennard-Jonesium, the results were found to be in good agreement with previous simulation studies using other approaches.

## 1 Introduction

Knowledge of phase equilibrium properties of solid systems is important in many scientific and technological areas. For example, in pharmaceutical production and formulation, this information is key to polymorphism control in the manufacture of drug compounds, which is a critical issue as the bioavailability can vary between polymorphs.^1^ Determination of thermo-physical properties of solid phases has been an important endeavor in molecular simulation, which has led to the development of various algorithms including the cell model,^2−4^ the Einstein crystal method,^5−7^ the phase switching Monte Carlo technique,^8−10^ and a modified Gibbs ensemble Monte Carlo (GEMC) approach.^11^ Some of these methods require free energy calculations. For example, in the Einstein crystal method, free energies of solids are determined via thermodynamic integration with the use of a λ parameter to link the real system to an Einstein crystal for which the free energy is known. The GEMC^12−14^ approach was developed specifically for direct calculation of phase coexistence properties, in which particle exchange is used to allow for the chemical potential equilibrium between the coexisted phases but would not succeed with the conventional setup for phase equilibrium calculation involving solids.^15^ The modified version uses a solid slab surrounded by vapor to overcome this issue and has been found successful for quite a few systems.^11,16,17^ The use of a solid slab makes it less straightforward to employ analytical tail corrections than the conventional GEMC setup. Without tail corrections, a relatively large cutoff is typically used, which requires a system containing thousands of particles.

All methods developed so far include the use of bulk-phase systems. In this work, it is demonstrated that one can use a cluster setup to study phase coexistence properties involving solids. This development was inspired by the open-surface setup in the modified GEMC technique to allow for efficient particle transfer. It was also motivated by many previous studies that focused on vapor–liquid nucleation where it was found that one can use the size dependencies of the formation free energies of the clusters to extrapolate the bulk-phase properties such as the chemical potential at coexistence and the surface tension.^18−25^ No systematic study has been performed to examine the accuracy of the bulk-phase properties extrapolated from this approach as extremely high-quality results are required, which are often lacking except for a few systems.

In our previous work, the aggregation-volume-bias Monte Carlo (AVBMC)^26,27^ method was used to allow for efficient particle exchange between the vapor phase and the cluster phase. However, when the conventional AVBMC method was employed, liquid-like clusters were formed at the beginning even at conditions far below the triple point.^18^ The free energies obtained for these liquid-like clusters can be only used to extrapolate the phase properties of the corresponding liquid phase, which is metastable at *T* < *T*_m_. In order to avoid the formation of liquid-like clusters, an extension of the AVBMC method is introduced here, in which the insertion of particles is targeted directly toward those crystal lattice sites. Specifically, the insertion volume used to be defined as a spherical volume centered around a given target molecule is now restricted to be around each of the crystal lattice sites near a given target molecule. The extended algorithm is applied to a Lennard-Jones system for which high-quality bulk-phase properties are available for comparison, including the surface tension and the triple point. Section 2 presents the details of this extended algorithm as well as the molecular models and simulation details of this study. The results of the simulations are presented and discussed in Section 3, and Section 4 provides concluding remarks.

## 2 Methods

**Figure 1:**
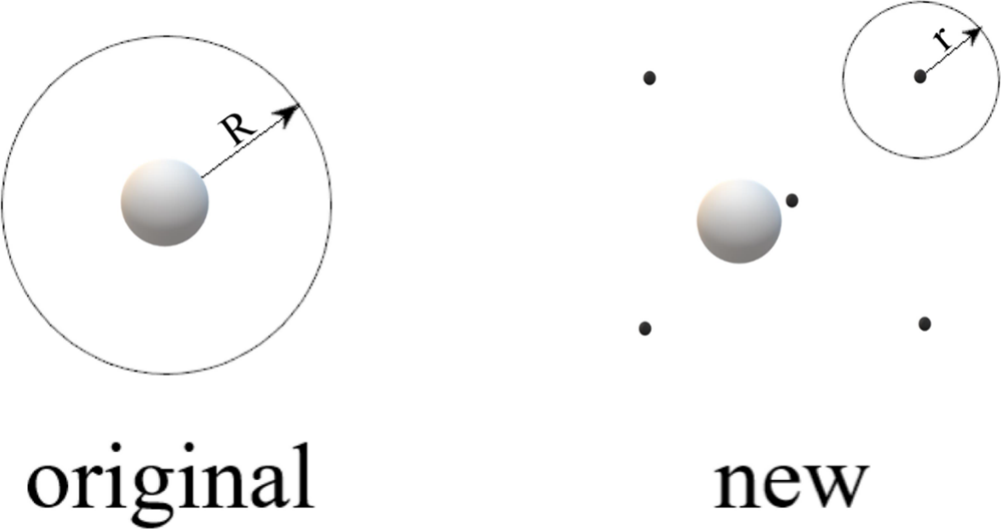
Schematic drawing of the volume used for particle insertion. In the original AVBMC scheme, the volume used for particle insertion is centered on a target particle, whereas in the lattice-based AVBMC scheme, the volume used for particle insertion is centered on one of the lattice sites neighboring to the lattice site that the target particle belongs to.

AVBMC^26,27^ was originally developed to allow for efficient sampling of the formation or destruction of clusters in strongly associating fluids. It was inspired by the particle swap moves employed in the GEMC^12−14^ technique. While in GEMC, particle swap moves are used to equilibrate the chemical potentials between different bulk phases, in AVBMC, these moves are used to equilibrate the chemical potentials between the vapor phase and the cluster phase. Such direct particle transfer can greatly speed up the sampling of both cluster growth and destruction. Namely, one can pick a particle from the vapor phase and directly place it into the cluster phase to avoid the long time-scale diffusion process due to the large spatial separation between the cluster and the monomers. Since an entropic (or volume) biasing factor is introduced here, to satisfy the detailed balance condition, this factor would be part of the acceptance rule used for both particle insertion and deletion. For the particle deletion move, this entropic (or volume) factor would compensate for the energetic factor due to a particle leaving the cluster, leading to an improved acceptance rate. It should be noted that a major difference between GEMC and AVBMC is on the volume used for particle swap moves. While the former uses the volume of the entire simulation box, AVBMC employs a more flexible choice of a local volume, typically, a spherical volume centered on a target molecule (see Figure 1), which allows for intrabox swap moves.

### 2.1 Lattice-Based AVBMC

Also shown in Figure 1 is the general setup of the modified version of the AVBMC method. As mentioned in the Introduction, when the original algorithm is used, the clusters formed are disordered even at a temperature below the melting point. In the modified AVBMC, the volume used for particle exchange moves is now restricted to be near the lattice sites to discourage the formation of disordered structures. For simplicity, it is defined as a spherical volume (*V*_in_) centered around each lattice site. This method requires a solid lattice setup where each particle in the cluster is assigned to a specific lattice site. Each site can have no more than one particle. The lattice replicates as the cluster grows. A new cluster criterion is used to define these solid clusters, that is, two particles which occupy two adjacent lattice sites are considered neighboring to each other and each particle needs to be bound with a certain distance specified by the parameter *r* (see Figure 1) from its nearest lattice site. Starting with a given configuration containing *N* molecule in the cluster, a five-step procedure is used for the particle swap move employing the modified AVBMC algorithm between the gas phase and the cluster phase:

1. the move type is randomly picked as either insertion or deletion with equal probabilities;
2. a particle, say *j*, is randomly picked from the cluster as the target for the swap move;
3. for an insertion move, one of the *N*_vac_ vacant lattice sites is randomly picked (move is automatically rejected if no vacant sites are available), and then a new particle is randomly inserted inside a spherical region centered around this site; for a deletion move, one of the *N*_in_ neighbors is randomly picked (move is automatically rejected when particle *j* does not have any neighbors or when the remaining particles no longer satisfy the cluster criterion);
4. the potential energy difference, Δ*E*, is computed due to this move; and
5. the new configuration is accepted with the following probability at a given inverse temperature β (assumed that the grand canonical ensemble is used, where the cluster is coupled with an ideal gas reservoir at a given chemical potential μ):
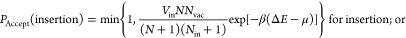

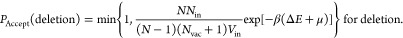

### 2.2 Molecular Models and Simulation Details

All simulations were performed for a Lennard-Jones system. Formation free energies of both liquid and solid clusters with a size up to 8000 particles were calculated using the grand canonical ensemble in which the cluster was thermodynamically connected with an ideal gas-phase reservoir at a certain chemical potential or density. For these simulations, all interactions were included. Simulations for solid clusters are performed using a face-centered cubic lattice. To determine the spacing between two adjacent lattice sites, isobaric-isothermal ensemble simulations were carried out using 864 particles with a spherical cutoff at 4.75 σ and with analytical long-range corrections at the corresponding temperatures and pressures obtained from previous GEMC simulations.^18^ The spacing between two adjacent lattice sites was found to be 1.128 at *T* = 0.6. Simulations were carried out with various values of *r* used to define the spherical volume centered around the target site (see Figure 1) to examine how the simulation results would depend on this parameter. In addition to AVBMC swap moves between the cluster and the vapor phase, these moves are also used to allow a randomly picked particle in the cluster phase to jump from one position to another, picked uniformly, within each spherical volume around the lattice site that the particle belongs to. The acceptance rule for such moves is governed only by the Boltzmann factor associated with the energy change between the proposed and the old configuration. These “intrasite” swap moves function as translational moves and should automatically satisfy the additional condition required by the solid cluster criterion (i.e., each particle cannot be found more than *r* distance away from its lattice site). These two types of moves are picked randomly with equal probabilities. The acceptance rates for insertion and deletion moves range from 6% for a cluster size of 100 to 1% for a cluster size of 8000 when using *r* = 0.5 at *T* = 0.6. For simulations of liquid clusters, half of the moves are spent on regular translational moves with a maximum displacement of 0.2 for all clusters. The acceptance rates for AVBMC swap moves range from 4% for a cluster size of 100 to 0.8% for a cluster size of 8000 when using *R* = 1.5 at *T* = 0.6. For comparison, the acceptance rate for particle swap moves is 0.002% from a bulk-phase GEMC simulation containing 2200 particles at this temperature which also use ∼50% of the moves on both translation and swap and 0.01% of moves on volume exchange. The significantly higher acceptance rate found for AVBMC moves for the cluster simulations is due to the presence of the surface.

Umbrella sampling^28^ is used to speed up the convergence of the formation free energy for clusters by employing a biasing potential that is negative to the estimated formation free energy so that all clusters are evenly sampled in the simulation. For clusters within a relatively small size range, this convergence can be done rather quickly using a self-adapting iterative procedure. Namely, the formation free energy results estimated with a given initial biasing potential are used to guess the biasing potential to be implemented in the next iteration run until even sampling is achieved. When the quality of the free energy is good for a large enough size range (for clusters containing up to 50 particles), one can use a linear extrapolation scheme based on the size dependency of the cluster free energy prescribed by the classical nucleation theory (CNT)^29−35^ to project what the formation free energies would be for larger clusters (or what biasing potential values are needed so that they can be evenly sampled in the simulation). It is the same scheme that is used for extrapolating the chemical potential of an infinitely large cluster, that is, bulk phases. The basic idea is that the formation free energy of a cluster containing *n* particles, Δ*G*(*n*), comes from two components: a bulk term which is proportional to the size of the cluster (or *n*) and a surface term which is proportional to the surface area (or *n*^2/3^). By plotting δΔ*G* (= Δ*G*(*n*) – Δ*G*(*n* – 1)) as a function of *n*^2/3^ – (*n* – 1)^2/3^, a straight line is obtained with an intercept governed by the chemical potential difference between a cluster of an infinite size (i.e., either bulk solid or liquid) and the mother phase (which would be the ideal gas phase used in the grand canonical ensemble). The slope, *s*, on the other hand, can be related to the surface tension value γ, that is, *s* = (36π/ρ^2^)^1/3^γ, where ρ refers to the density of the bulk phase.

This linear interpretation requires cluster formation free energies with unprecedented accuracy. It does not require the calculation to be performed for clusters of all sizes within a certain range, which is typically done for the vapor–liquid nucleation calculations, as the calculation of δΔ*G* needs only two adjacent Δ*G* values. For computational efficiency, free energy calculations are performed sparsely over a large cluster size range, that is, for a few clusters near a size of 100, and then a few clusters near 200, etc., with each cluster sampled at least 10^10^ times so that the extrapolation with linear fits to these data can lead to an extremely precise estimate of both the intercept and the slope. The cost of the simulation scales linearly with the size of the cluster, from about 3 h of CPU times per cluster at a size of 100 to 280 h per cluster at a size of 8000 using a 2.8 GHz Intel Ivy Bridge-EP processor. The errors are estimated by dividing the simulations into five blocks.

## 3 Results and Discussion

### 3.1 Systematic Examination of the Bulk Property Extrapolation Scheme Used in This Work by Comparing to the Results Obtained Using Other Methods

**Figure 2:**
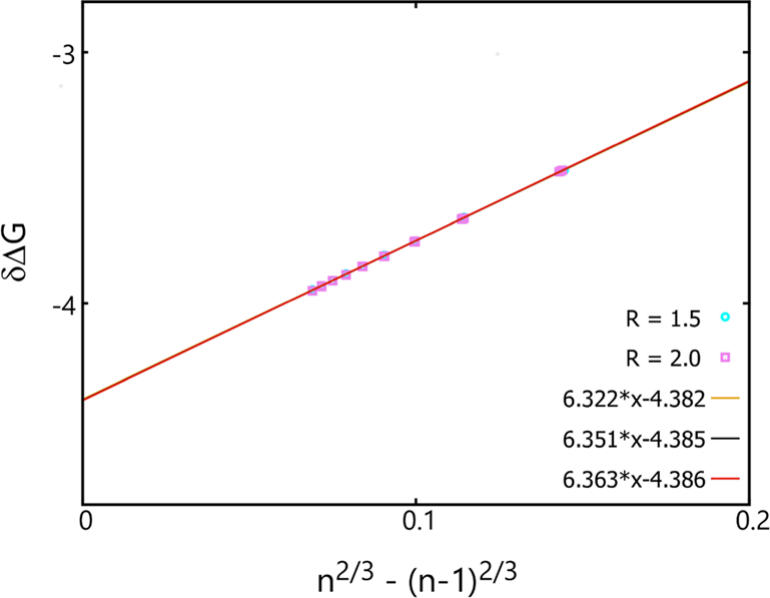
δΔ*G* (= Δ*G*(*n*) – Δ*G*(*n* – 1)) as a function of *n*^2/3^ – (*n* – 1)^2/3^ obtained using the original AVBMC algorithm and the original cluster criterion with *R* = 1.5 (cyan circles) or 2.0 (purple squares). Linear fits performed over the three cluster size ranges are shown only for the case with *R* = 1.5 as orange (100–300), black (400–600), and red (700–900).

Shown in Figure 2 are the δΔ*G* values obtained at *T* = 0.7 and at an ideal gas-phase density, *n*_v_ = 1, plotted as a function of *n*^2/3^ – (*n* – 1)^2/3^ using the original AVBMC scheme and an *R* value of 1.5 (the radius used to define the insertion volume and also the cluster criterion, see Figure 1), for clusters containing between 100 and 900 particles. When only a single linear fit is performed on this entire size range, it would yield a straight line with a slope value of 6.344 and an intercept value of −4.385. As mentioned in the previous section, the intercept obtained via this linear fit can be used to predict the chemical potential difference between the bulk liquid phase and the ideal gas phase at *n*_v_ = 1 (which would have a chemical potential value of zero excluding the kinetic term). Thus, using this extrapolation scheme, a chemical potential value of −4.385 (excluding the kinetic term) is obtained for the bulk liquid phase, which compares well with a value of −4.370 ± 0.009 obtained previously from a GEMC simulation for a system containing 500 particles with a cutoff at 3.75 σ and tail corrections.^36^ Finite-size corrections^37^ would lower this value to reach a potentially better agreement. Indeed, a repeat of the GEMC run for a system containing 2200 particles with a cutoff at 5 σ and tail corrections yielded a chemical potential value of −4.384 ± 0.002. Using the slope value of 6.344 and the bulk liquid-phase density of 0.843 ± 0.001 obtained at this temperature via the additional bulk-phase simulations, a surface tension value of 1.170 is yielded, which compares well with a value of 1.182 ± 0.010 interpreted using a combination of finite-size scaling techniques and grand canonical transition-matrix Monte Carlo simulations for an infinite system,^38^ and is slightly larger than a value of 1.15 ± 0.02 using a conventional liquid slab setup with a large cutoff value at 6.5 σ and a system containing 2048 particles.^39^

To show how the bulk-phase properties interpreted from this scheme would depend on the system size, linear fits are also performed separately for the following three size ranges: 100–300, 400–600, and 700–900. As shown in Figure 2, there is only a small dependence of both the slope and the intercept on the system size. The interpreted surface tension values yielded from linear fits of δΔ*G* values to these three size ranges are 1.167 ± 0.001, 1.172 ± 0.003, and 1.173 ± 0.007. The interpreted chemical potential values for the bulk liquid yielded from these three linear fits are as follows: −4.382 ± 0.001, −4.385 ± 0.002, and −4.386 ± 0.003. Even with the smallest cluster size range, that is, between 100 and 300, one can obtain fairly accurate estimate of both surface tension and chemical potential values for the bulk phase.

To examine how the bulk-phase properties extrapolated from this scheme would depend on the radius used to define the insertion volume and the cluster criterion, additional simulations were performed using a large *R* value of 2. The δΔ*G* results obtained in these simulations are also shown in Figure 2, which are nearly identical to those obtained using *R* = 1.5. Similarly, linear fits are performed over the same three size ranges. The following surface tension values are obtained: 1.180 ± 0.001 (100–300), 1.175 ± 0.004 (400–600), and 1.175 ± 0.006 (700–900). The following chemical potential values are obtained: −4.391 ± 0.003 (100–300), −4.388 ± 0.002 (400–600), and 4.388 ± 0.002 (700–900). These results are in excellent agreement with those found in the literature,^36−39^ which again supports the use of this scheme on small clusters for accurate interpretation of bulk-phase properties.

### 3.2 Comparison of the δΔ*G* Results Obtained Using the Original and the New AVBMC Scheme

The use of δΔ*G* plots in the interpretation of bulk-phase properties has so far been restricted to the liquid phase. As shown in previous simulations, using the original AVBMC algorithm only liquid clusters are formed even at conditions far below the triple point (and the liquid-to-solid transition occurs after a sufficiently large cluster is formed—such a two-step nucleation mechanism has been supported by both theories^18,40,41^ and experiments^42−44^). Linear fits of these liquid cluster data would provide the bulk liquid-phase properties but not the solid phase. This new lattice-based AVBMC scheme is developed to specifically restrict the cluster growth to follow a path to a solid structure right from the beginning, thereby opening up a new avenue of using these solid clusters in obtaining the properties of bulk solid.

**Figure 3:**
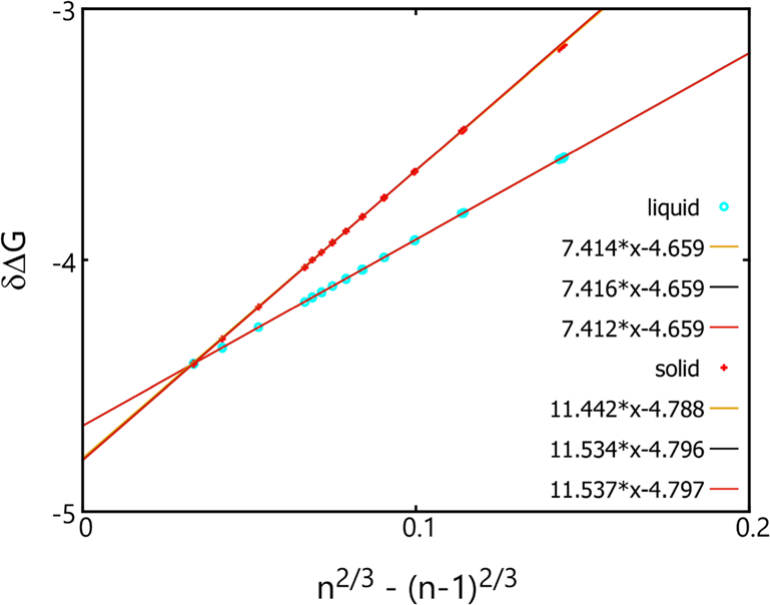
δΔ*G* (= Δ*G*(*n*) – Δ*G*(*n* – 1)) as a function of *n*^2/3^ – (*n* – 1)^2/3^ obtained using the original AVBMC algorithm and the original cluster criterion with *R* = 1.5 (cyan circles) or using the new lattice-based AVBMC algorithm and the new cluster criterion with *r* = 0.25 (red pluses). Linear fits performed over the three cluster size ranges are shown as orange (200–500), black (600–900), and red (1000–8000).

Shown in Figure 3 are the δΔ*G* values obtained at *T* = 0.6 (which is below the triple point known for this system) and at an ideal gas-phase density, *n*_v_ = 1, plotted as a function of *n*^2/3^ – (*n* – 1)^2/3^ using the original AVBMC scheme and an *R* value of 1.5 compared to those obtained using the lattice-based AVBMC scheme and an *r* value of 0.25. Linear fits of these data are performed over three cluster size ranges: 200–500, 600–900, and 1000–8000. For the data obtained using the original AVBMC scheme (denoted as “liquid” since clusters formed are liquid-like even though the temperature is below the triple point), both the slope and the intercept obtained from these three linear fits are consistent with each other within the uncertainties. Using the slope values and the bulk liquid-phase density of 0.882 ± 0.001 obtained at this temperature via the bulk-phase simulation, a surface tension of 1.410 is obtained at this temperature, which is slightly larger than a value of 1.397 ± 0.008 obtained using a conventional liquid slab setup and a finite system with around 2000 molecules,^18^ which is consistent with the finding at *T* = 0.7 (see Section 3.1.). The linear interpretation over all three cluster size ranges (including a significantly larger cluster size range, 1000–8000, than the previous section) all leads to the same chemical potential value of −4.659 (all with an error less than 0.001), which is in excellent agreement with a value of −4.657 ± 0.002 obtained from a GEMC simulation for a system containing 2200 particles with a cutoff at 5 σ and tail corrections.

For the solid clusters, the linear fit obtained for the smallest cluster size range (200–500) exhibits a small but noticeable difference on both the slope and the intercept value, while the results obtained for the other two larger cluster size ranges (600–900 and 1000–8000) agree well with each other within the statistical uncertainties. All three linear fits produce a lower intercept value than −4.659 obtained for the liquid clusters, suggesting that the bulk solid phase is more stable than the bulk liquid phase, although the δΔ*G* values obtained for these solid clusters are significantly higher than those found for the liquid clusters within this cluster size range. This agrees with the two-step crystal nucleation mechanism found previously from both theoretical and experimental work^18,40−44^ that without this lattice constraint, nucleation would automatically proceed with the formation of an initially liquid-liked cluster, which is more stable than the solid cluster, followed by a crystallization inside this liquid-like cluster after passing a certain size threshold when the solid cluster becomes more stable.

### 3.3 Dependence of the δΔ*G* Results Obtained Using the New AVBMC Scheme on the Radius Used to Define the Insertion Volume

**Figure 4:**
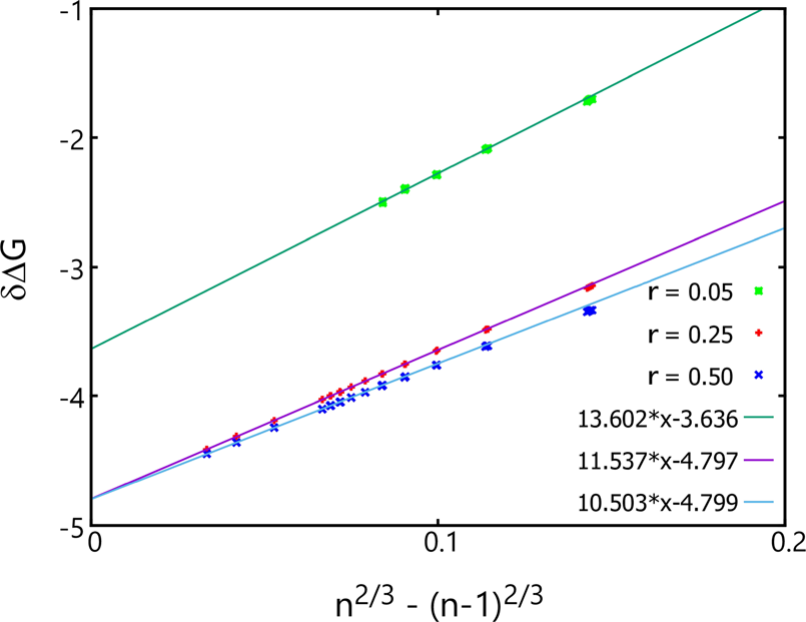
δΔ*G* (= Δ*G*(*n*) – Δ*G*(*n* – 1)) as a function of *n*^2/3^ – (*n* – 1)^2/3^ obtained using the new lattice-based AVBMC algorithm and the new cluster criterion with *r* = 0.05 (green), 0.25 (red), and 0.50 (blue). Only linear fits performed over the largest cluster size range are shown.

**Figure 5:**
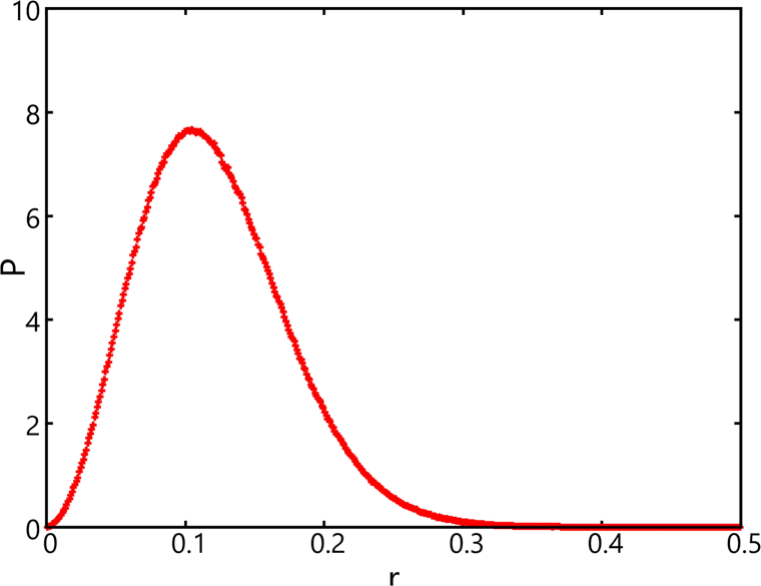
Normalized probability of finding a particle as a function of its distance away from its lattice position in the solid phase.

Shown in Figure 4 are the δΔ*G* values plotted as a function of *n*^2/3^ – (*n* – 1)^2/3^ using the lattice-based AVBMC scheme with an *r* value ranging from 0.05 to 0.5. Compared to the liquid cluster data shown in Figure 2, the δΔ*G* values obtained for the solid clusters are much more sensitive to this radius parameter that is used to define the insertion volume. In particular, the set of data obtained using *r* = 0.05 differs dramatically from the other two sets obtained with *r* = 0.25 or 0.5. This result can be understood in light of the data presented in Figure 5, obtained from a bulk-phase simulation, which show that particles can move much further away from the lattice position with most particles being found at around 0.1 off its lattice site. The chemical potential value interpreted from the linear fits to the δΔ*G* data obtained with *r* = 0.05 would be for an entirely different solid model. On the other hand, linear fits to the other two sets of δΔ*G* data (over the cluster size range between 1000 and 8000) produce two intercept values, −4.7966 ± 0.0002 (*r* = 0.25) vs −4.7990 ± 0.0006 (*r* = 0.5), that are very close to each other. The small difference could be due to the fact that a use of *r* = 0.25 may not be large enough as in the bulk solid a small fraction of particles can be found outside that range. Given that the difference between these two sets of data is more noticeable on the slope (related to the surface tension), a use of *r* = 0.25 instead of 0.5 appears to affect more the interface than the bulk.

### 3.4 Interpretation of the Triple Point

Using the intercepts obtained from the linear fits to the δΔ*G* plots obtained at *T* = 0.6 for both liquid and solid clusters, the chemical potential values are estimated to be −4.6594 ± 0.0009 for the bulk liquid phase and −4.7990 ± 0.0006 for the bulk solid phase (or 0.1396 lower than the liquid phase). Both are at the same pressure condition using the current setup (the liquid clusters, although isolated, can be viewed to be surrounded by a thin layer of saturated vapor phase above it with a sufficiently large *R*, whereas the lattice spacing for the solid clusters is determined from the isobaric-isothermal ensemble simulation under the saturated vapor pressure of the bulk liquid phase obtained from the GEMC simulation). Using the Gibbs–Helmoholtz^45^ equation and the enthalpy data obtained from the isobaric-isothermal ensemble simulations, the melting point (at which the chemical potential is equal between these two bulk phases) was estimated to be about 0.6897 ± 0.0010 at this pressure. Starting from this point with the Clapeyron equation,^45^ the solid–liquid coexistence curve can be constructed. Combined with the liquid–vapor coexistence curve, the triple point can be located, which is slightly higher, at 0.6898 ± 0.0010. The triple point which has been reported for this system scatters from 0.661 to 0.7085.^11,46−53^ Schultz and Kofke^46^ have recently carried out an extensive investigation on this system by computing the properties in the limit of an infinite cutoff radius and in the limit of an infinite number of atoms and found the triple point to be 0.69455 ± 0.00002. The difference between their estimated triple point and this work can be translated into a difference of <0.007 in terms of the chemical potential value. For solid clusters, the chemical potential value interpreted from linear fits of the δΔ*G* plots can be viewed as an upper bound for the bulk phase as it keeps decreasing as a larger range of clusters are used (see Figure 3).

### 3.5 Further Discussion

Both surface tension and chemical potential are among those most challenging properties to be determined by molecular simulations. For example, an extensive recent review of the literature data on the thermodynamic properties of the Lennard-Jones fluid, all calculated using the bulk-phase systems, has shown the precision of the surface tension to be ±4% vs ±0.2% for the saturated liquid densities.^54^ One reason for the large spread on the surface tension is due to the truncation of intermolecular interactions or the treatment of the long-range corrections.^39,55^ For the bulk-phase setup, the general consensus is that accurate determination of the surface tension requires either a large cutoff or an explicit inclusion of long-range corrections. It is encouraging that the surface tension extrapolated from the current cluster-based approach to the bulk limit would fall within that ±4% spread (or within 1% from the prediction by the correlation function, i.e., eq 11 in ref (54).), even when a relatively small cluster size range is used. This is partly because this extrapolation scheme explicitly considers the dependency of the surface free energy on the cluster size, that is, proportional to the surface area or *n*^2/3^ (see the *x*-axis used in Figures 2–4).

**Figure 6:**
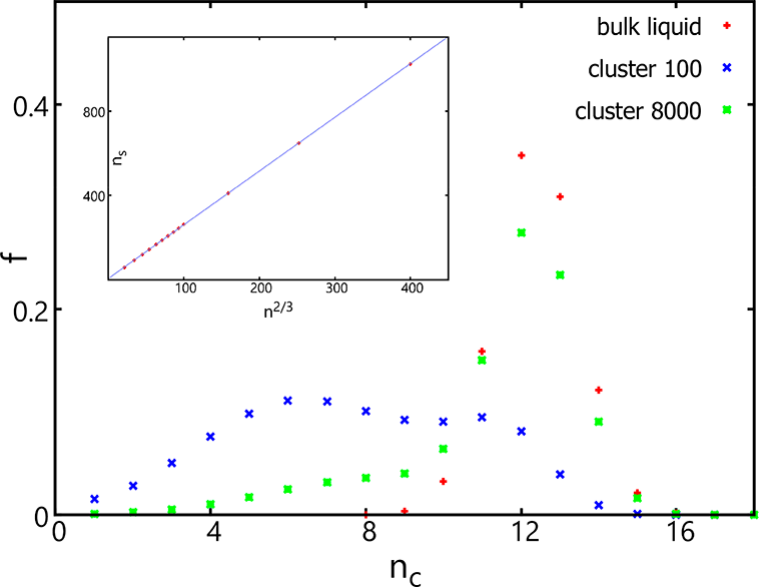
Fraction of particles as a function of the coordination number (or the number of neighbors) obtained for the bulk liquid (red) and clusters containing 100 (blue) or 8000 (green) particles using the original AVBMC scheme and the original cluster criterion with *R* = 1.5. The inset shows how the number of surface particles (defined to be those that have fewer than 9 neighbors) scales with the *n*^2/3^ term used in plotting the δΔ*G* results.

To gain further insights, the distribution of coordination numbers was analyzed for clusters of different sizes and compared to that of the bulk phase (see Figure 6). Whereas this distribution shows a single peak at a coordination number of 12 for the bulk phase, it is bimodal when clusters are small. For example, at a size of 100, this distribution shows a second peak at a coordination number of 6. With the increase of the cluster size, this peak diminishes and becomes a shoulder (see the distribution for clusters containing 8000 particles). If assuming those particles with lower coordination numbers (i.e., below 9) are surface particles, the number of surface particles scales reasonably well with *n*^2/3^ (see the inset in Figure 6).

**Figure 7:**
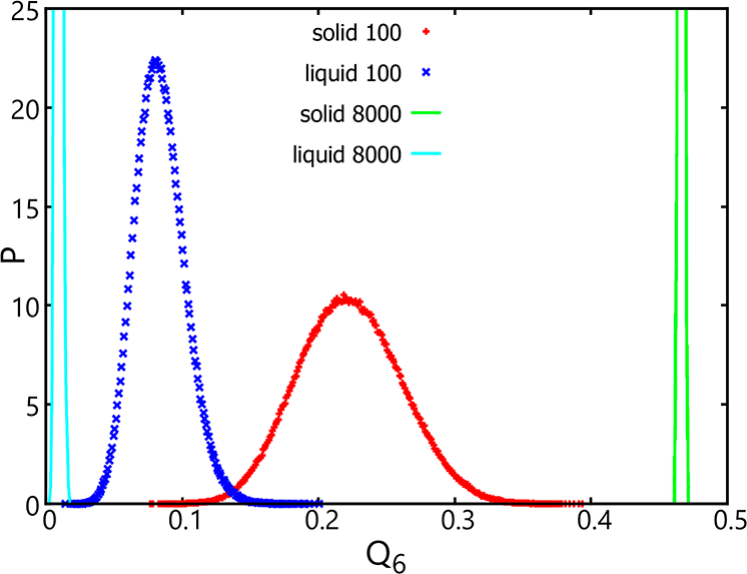
Normalized probability distribution of the Steinhardt^56^ order parameter *Q*_6_ for clusters containing 100 particles using the new AVBMC scheme and the new cluster criterion with *r* = 0.5 (red pluses) vs the original AVBMC scheme and the original cluster criterion with *R* = 1.5 (blue crosses). Green and cyan lines represent the results obtained using these two methods for clusters containing 8000 particles.

For the Lennard-Jones system, it has been shown that even at a temperature far below the melting point, clusters formed at the beginning are liquid-like.^18^ When clusters are large enough, the crystalline structure would become more stable, but the probability to observe the liquid-to-crystalline transition is exceedingly low because of the large barrier separating these two structures. For example, the crystal nucleation barrier was estimated to be 63 *k*_B_*T* at *T* = 0.6,^18^ or the probability to cross this barrier is on the order magnitude of 10^–28^. Thus, without an explicit biasing sampling along the order parameter that can be used to characterize the liquid-to-crystalline transition, the clusters using the original AVBMC method would remain liquid-like given the current computational resources, which is confirmed by the analysis of the cluster structures in terms of the Steinhardt^56^ order parameter *Q*_6_ (see Figure 7). In particular, at a cluster size of 8000 when the δΔ*G* value was found similar between the liquid and solid clusters, the distribution of the *Q*_6_ values is entirely different. For liquid clusters at this size, this distribution is narrowly centered at a low value of 0.009, which is expected when the structure is disordered. In contrast, the distribution for the solid clusters is narrowly centered at a large value of 0.468, which indicates that particles toward the surface of solid clusters are fairly ordered, due to the restriction of every particle in the cluster to be within *r* = 0.5 from its lattice site.

Such a restriction may make it difficult to use this approach to model accurately the surface of solid systems (such as surface tension and surface free energy) as the outer layer tends to be liquid-like, which has been shown previously in the crystal nucleation study for large LJ clusters.^18^ By reducing the *r* parameter, one would expect that the surface becomes even more solid-like, which explains why the slope of the δΔ*G* plots increases when *r* decreases from 0.5 to 0.05 (see Figure 4). Because of the sensitivity of the free energy results to the *r* parameter, it would be also difficult to use these free energy results to quantify the size-dependent melting point for clusters, which is defined by the point when the value of Δ*G* is equal between the liquid and the solid cluster. However, without this restriction, the cluster would automatically melt and become liquid-like even starting with a purely solid cluster unless the size of the cluster is sufficiently large and/or the temperature is low enough to stabilize the solid cluster. Such a barrierless melting is presumably initiated by the liquid-like surface layer, that is, without this seeding layer when melting occurs in the interior of a solid phase, it may also need to cross a nucleation barrier, analogous to the bubble nucleation inside a liquid phase. Once fully melted, the reverse process is rather more challenging to handle by molecular simulation as the liquid-to-solid transition always needs to cross a barrier. When the barrier is too low, the system may become amorphous due to the ramification of multiple crystallites.^18^ When the barrier is too high, the free energy calculation would need to be performed along additional order parameters (such as Q_6_) and biasing sampling along these order parameters may still lead to the ramification issue. On the other hand, the presence of the crystal nucleation barrier makes it possible to extrapolate the liquid properties far below the melting point through clusters defined by the conventional cluster criterion.

The lattice-based technique presented here further explores this idea of whether one can employ solid clusters (defined by a new cluster criterion) to extrapolate the properties of solid systems as it sounds theoretically plausible since the interior structure of the cluster can be made as similar as possible to that of the bulk solid with the choice of an appropriate lattice spacing and a sufficiently large *r*. The simulation results presented here appear to be supportive of this idea.

## 4 Conclusions

A latticed-based AVBMC method has been developed to enable the study of the properties of solid systems. This method differs from the original one in the volume used for particle swap moves. Instead of using a spherical volume centered around a target molecule, it uses a volume that is strictly centered around the solid lattice site. This ensures that all clusters formed are solid-like. Using both the original and the new AVBMC method, the formation free energies are calculated for both liquid and solid clusters, which are then used to extrapolate bulk-phase information such as surface tension and chemical potential through linear fits of the δΔ*G* plots. It has been found that such linear fits can lead to very accurate estimates of both surface tension and chemical potential values for the bulk phase. The chemical potential values obtained for both liquid and solid, combined with the Gibbs–Helmholtz equation, are used to determine the melting point. By tracing the coexistence lines with the Clapeyron equation, the triple point is estimated. For a Lennard-Jones system, a triple point of at least 0.6898 was found using this approach.

Finally, this method can be further improved by combining with more sophisticated biasing schemes, such as those introduced in ref (57) to preferentially select the interface region for particle swap moves. This method can also be combined with configurational-bias Monte Carlo^58−63^ to enable the study of phase properties of more complex molecules. It can be further extended to other types of lattices to investigate the relative stabilities of different crystal structures.
